# Rectus Sheath Hematoma: An Unfortunate Consequence of Novel Anticoagulants

**DOI:** 10.5811/westjem.2015.2.25631

**Published:** 2015-04-02

**Authors:** Kimberly Stillman, Jesse Kellar

**Affiliations:** Lakeland Regional Medical Center, Department of Emergency Medicine, St. Joseph, Michigan

## INTRODUCTION

A 76-year-old male presented to the emergency department complaining of intense abdominal pain. He reported one week earlier an upper respiratory illness with violent coughing spells. Past medical history included recent percutaneous coronary intervention for a myocardial infarction 6 months prior where he received three drug-eluting stents and was subsequently discharged home on Prasugrel (Effient) and Aspirin.

**Figure 1:**
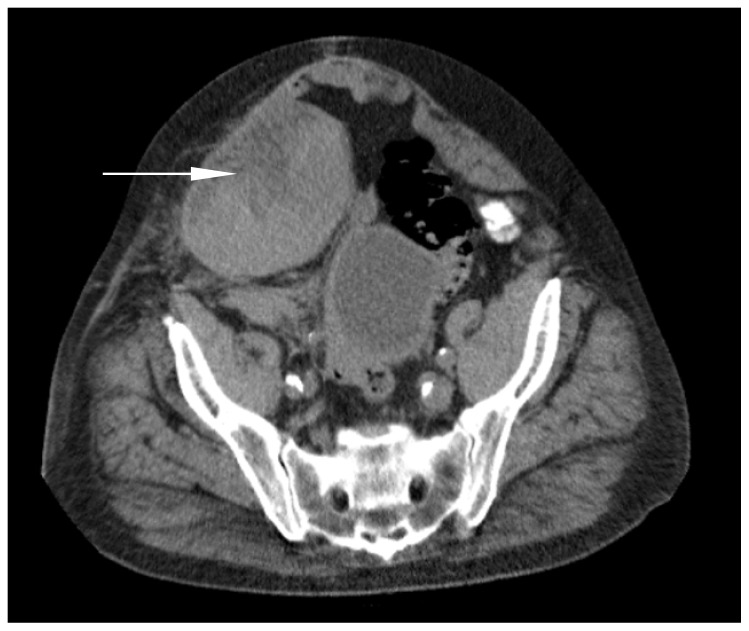
Axial view computed tomographic scan revealing hematoma within the rectus sheath (arrow).

**Figure 2:**
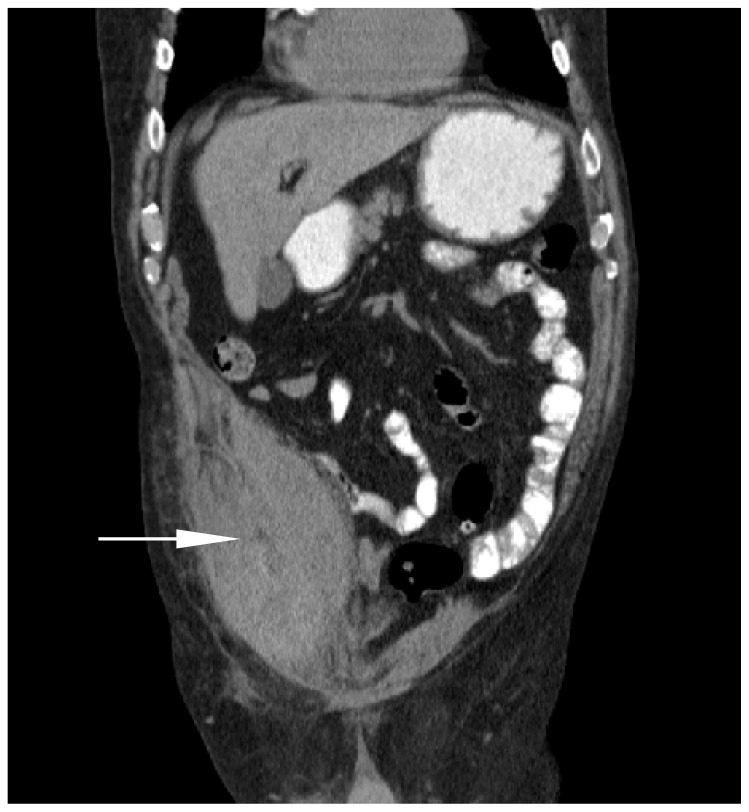
Coronal view computed tomographic scan revealing hematoma within the rectus sheath (arrow).

Physical exam revealed a large tender right lower quadrant mass with areas of ecchymosis appreciated over the supra-pubic and right lower abdominal region. Abdomen was otherwise soft and non-distended. Basic laboratory tests were noted to be within normal limits. A computed tomography (CT) of the abdomen and pelvis was performed and revealed a 12cm rectus sheath hematoma in the right lower quadrant (Figures 1 and 2).

## DIAGNOSIS

Rectus sheath hematomas (RSH) are often misdiagnosed and overlooked as a cause of acute abdominal pain. It has been estimated that RSH account for 1.5–2% of unexplained abdominal pain in hospitalized patients,^1^ but with the widespread use of newer agent anticoagulants this number is likely on the rise.

RSH result from the accumulation of blood in the rectus sheath, secondary to disruption of the blood vessels that course through it. The most common inciting factors are direct trauma, strenuous straining (e.g. coughing, exercise, vomiting), and anticoagulants.^2^ Large hematomas are more likely to occur in patients who have disruption of one of the epigastric arteries in combination with anticoagulant use.

The mortality rates associated with RSH can be as high 25% for those patients on anticoagulation drugs^3^ and is due to a delay in diagnosis as symptoms are often non-specific. Not all patients will have a visible hematoma on physical exam at time of presentation, often leading to further delays in diagnosis. The diagnostic modality of choice is CT of the abdomen and pelvis, which is believed to be 100% sensitive^4^ for RSH.

Management of RSH is dependent on the grade of hematoma that encompasses the size, degree of anti-coagulation, and the patient’s hemodynamic status. Low-grade RSH can usually be managed with conservative treatment. Higher-grade hematomas require more aggressive treatment including blood products, reversal of anticoagulation, and in certain cases surgical evacuation.^2^,^5^

Our patient was treated conservatively with Desmopressin and was discharged without additional complications.
